# Size and distribution of the iron oxide nanoparticles in SBA-15 nanoporous silica via SANS study

**DOI:** 10.1038/s41598-019-52417-w

**Published:** 2019-11-01

**Authors:** Adriana Zeleňáková, Pavol Hrubovčák, Ondrej Kapusta, Norbert Kučerka, Aleksander Kuklin, Oleksandr Ivankov, Vladimír Zeleňák

**Affiliations:** 1 P. J. Šafárik University, Department of Solid State Physics, Košice, 041 54 Slovakia; 2 Joint Institute for Nuclear Research, Frank Laboratory of Neutron Physics, Dubna, 141980 Russia; 3 Comenius University in Bratislava, Department of Physical Chemistry of Drugs, Bratislava, 832 32 Slovakia; 4 Moscow Institute of Physics and Technology, Institutsky per. 9, Dolgoprudny, Moscow Region 141700 Russia; 5 P. J. Šafárik University, Department of Inorganic Chemistry, Košice, 041 54 Slovakia

**Keywords:** Structure of solids and liquids, Physics, Applied physics, Condensed-matter physics

## Abstract

Structural characteristics of nanocomposite series consisting of iron oxide nanoparticles (NPs) embedded in the regular pores of amorphous silica matrix (SBA-15) were investigated by means of small angle neutron scattering (SANS). By virtue of unique neutron properties, insight into the inner structure and matter organization of this kind of systems was facilitated for the first time. Based on rigorous experimental support, fundamental model describing the neutron scattering intensity distribution was proposed by assuming general composite structural features. Model application to SANS data confirmed the presence of iron oxide NPs in the body of examined matrices, providing additional information on their shape, concentration and size distribution. Scattering superposition principle employed in the model conception allows for tailoring its fundamental characteristics, and renders it a potent and versatile tool for a wide range of applications.

## Introduction

It has just been two decades since the synthesis of revolutionary periodic nanoporous material SBA-15 on the basis of amorphous silica was reported by Zhao *et al*.^1^. The famous quotation “Space–the final frontier” adopted by Davis in 2002^2^ as a metaphor expressing great expectations of future importance of ordered nanoporous systems turned out not pathetic at all. Extraordinary properties inherent to porous silica solids, e.g. high active surface area, regular and perfectly ordered pores along with chemical stability, thermal durability and biocompatibility immediately excited scientific community which was aware of their enormous application potential^1–4^.

Even after 20 years, the interest in revealing the vast utilization possibilities of these systems has not faded out. On the contrary, employment of modern scientific methods allowed for their more profound and extensive investigation on nano and atomistic scales. Consequently, detailed description along with principal understanding of fundamental properties of these systems have led to the development of novel advanced materials. Nanoporous silica serves there a hosting matrix, feasible to introduce a wide variety of features according to the desired area of application.

At the very beginning, only the benefits of enormous sorption potential inherent to hollow matrices were exploited. However, right after handling appropriate chemical methods for matrix modification, the idea of multifunctional porous nanosystems turned into reality. Brand new nanocomposites were designed and fabricated, soon finding their niche in catalysis^5^, cryomagnetic refrigeration^6^, engineering (sensors)^7^ or environmental industry^8^. However, with current advances in nanoscience, biophysics and medicine, the most promising utilization of such nanocomposites appears in the biomedicine^9^.

Martín-Saavedra *et al*.^10^ reported on the biocompatibility of composites containing *α*-Fe_2_O_3_ nanoparticles with diameter 5 nm embedded in an ordered mesoporous silica. Examined magnetic microspheres exhibited the ability of conducting magnetic hyperthermia upon application of a low-frequency alternating magnetic field. Cell culture experiments showed that, by adjusting the amount of magnetic microspheres and the time of exposure to the field, heat treatments of mild to very high intensities could be achieved^10^. Recently, mesoporous silica-based bioactive glasses were investigated by Vallet-Regí *et al*.^11^ showing another interesting properties. They were found to react chemically with the body fluids and generating the bone matrix. Gunawidjaja *et al*.^12^ inspected the local structures in improved kind of such bioactive glasses. Their pore walls comprise CaO–SiO_2_ phase primarily, while the inclusion of nanometre-sized calcium orthophosphate clusters are also present. The authors concluded that these systems are promising candidates for utilization in clinical fields. Namely, they can be employed in filling the osseous cavities, producing small parts for middle ear bone replacement and maxilofacial reconstruction, and in dental applications. Even though a variety of other progressive materials possessing such properties are known currently, their utilization is usually very specific and their potential for tailoring limited. Hence, the versatility of new porous silica nanocomposites provides a significant advantage over previous generation of smart materials. After an appropriate modification, they are able to provide several functions simultaneously which classifies these genuine smart materials as theranostatic systems^13^.

Further applications and ambitions to tune the novel nanocomposite materials require inevitably profound knowledge of their structural characteristics. Since the inner structure of silica matrix determines the geometry of objects forming inside the pores, it has a crucial impact on overall properties of the system. Our recent studies, where series of related systems have been analyzed and compared, revealed the influence of NPs concentration and silica dimensionality on adsorption/desorption capacity or magnetic characteristics of the composites in general^14^. Further, it has been demonstrated that size of NPs embedded in pores controls the magnitude of magnetocaloric effect^15^. It turned out that despite of the same wet-impregnation method employed during the particular matrix modification, several scenarios of nanoparticle formations are possible. Briefly, besides the regular creation of NPs within the pores, higher concentration of metallic precursor may bring about rapid precipitation triggering processes allowing the growth of particles on the surface of composite. Since there are not confined there, they can achieve sizes significantly larger than NPs embedded in the matrix body. In addition, the characteristics of particles loaded within the pores can also be regarded as open question. Due to peculiarities in symmetries of hosting matrices, nanoparticles’ shapes, size distribution, concentration and overall organization in composite exhibit rather high (and sometimes unexpected) variance. For instance, longitudinal regular pores (parallel and mutually isolated) of SBA-15 were intended to serve as a template for nanowires fabrication, but in practice it was found that the structures formed inside of pores while employing nanocasting method are small isolated objects rather than continuous nanorods. In the case of SBA-16 matrix with cubic symmetry, nanopores exhibit peculiar shapes resembling bottlenecks, they are mutually interconnected and organized in a kind of superlattice. Therefore, to hypothesize on the structure of objects precipitated inside the various matrices is very tricky. Finally, if we consider that the process of matrix modification is also sensitive to nature of metallic ions carried by precursors, one can imagine its vast complexity and wealth of various nanocomposites it may provide. To date, the evidence of the ample variability of systems based on matrices mentioned above is available^14,16–18^.

With the aim to describe and explain feasible properties of these materials, the majority of research groups are employing the conception of small particles embedded in the body of porous matrix. However, due to limits of standard structural analysis methods like X-ray diffraction (XRD), high resolution transmission electron microscopy (HRTEM) or sorption measurements, direct and plausible evidence on size, shape or distribution of the objects dispersed in the matrix is still absent. We attempted to employ neutrons as an alternative tool for nanocomposite structure determination. Their low absorption rates along with their very different nature of scattering interactions in comparison to the XRD allow us to scrutinize the matrix itself as well as the objects buried in its body. To the best of our knowledge, this is the first application of small angle neutron scattering (SANS) for this purpose. Obtained experimental data have been utilized in the extension of fundamental analytical model enabling a direct characterization of structural parameters of SBA-15 nanoporous silica as well as the objects embedded in it. It provides not only a direct evidence of the spherical shape of *α*-Fe_2_O_3_ nanoparticles introduced, but also information about their size distribution and concentration. Validity of the model has been corroborated by supplementary experimental results obtained from small angle X-ray scattering (SAXS), HRTEM and adsorption/desorption measurements. Although our model has been proposed for the particular system, its fundamental conception allows for its extension to other related structures, appearing thus as a potent tool for wider applications.

## Experimental Details

Small angle neutron scattering (SANS) measurements were performed at the IBR-2 pulsed reactor (with the pulse half-width of thermal neutrons being 320 *μs*, and frequency 5 Hz), that is run by Frank Laboratory of Neutron Physics, Joint Institute for Nuclear Research in Dubna (Russia). A small-angle time-of-flight axially symmetric neutron scattering spectrometer YuMO was used, which has two ring detectors of scattered intensity covering the scattering vectors *q* dynamical range 0.005–0.7 Å^−1^ (*q* = (4*π*/*λ*)*sinθ*, where *λ* is the neutron wavelength and 2*θ* is the scattering angle)^19^. Powdered samples were loaded in alumina cells and exposed to neutrons for approximately one hour at temperature 20 °C^20^. The scattering from empty cell was measured and subtracted from scattering of the samples. The averaged scattering patterns were corrected for detector efficiency absorption, solvent scattering, and instrumental background using the primary data treatment program SAS^21^.

The HRTEM micrographs were taken with JEOL 2100F microscope. Copper grid coated with a holey carbon support was used to prepare samples for the TEM observation. The bright-field TEM image was obtained at 200 kV.

X-ray diffraction measurements were carried out at the PETRA III facility in DESY, Hamburg (Germany). Powder samples were put into Capton capillaries and exposed to synchrotron radiation with energy 60 keV and corresponding wavelength *λ* = 0.0207 nm. Diffraction patterns were integrated and processed via FIT2D software and CeO_2_ was used as calibration standard.

## System Characterization

### Experimental support

**Figure 1:**
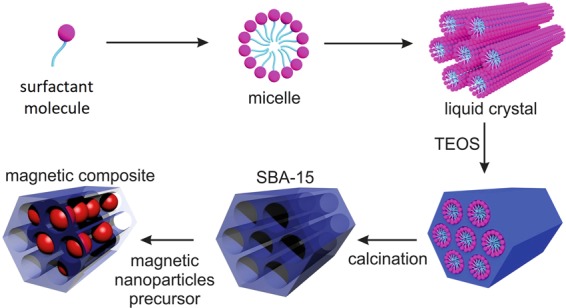
Illustration of the Fe_2_O_3_@SBA-15 nanocomposite preparation and structure organization. Polydisperse nanoparticles of Fe_2_O_3_ are randomly distributed in the longitudinal cylindrical pores of perfect hexagonal order within silica matrix.

**Figure 2:**
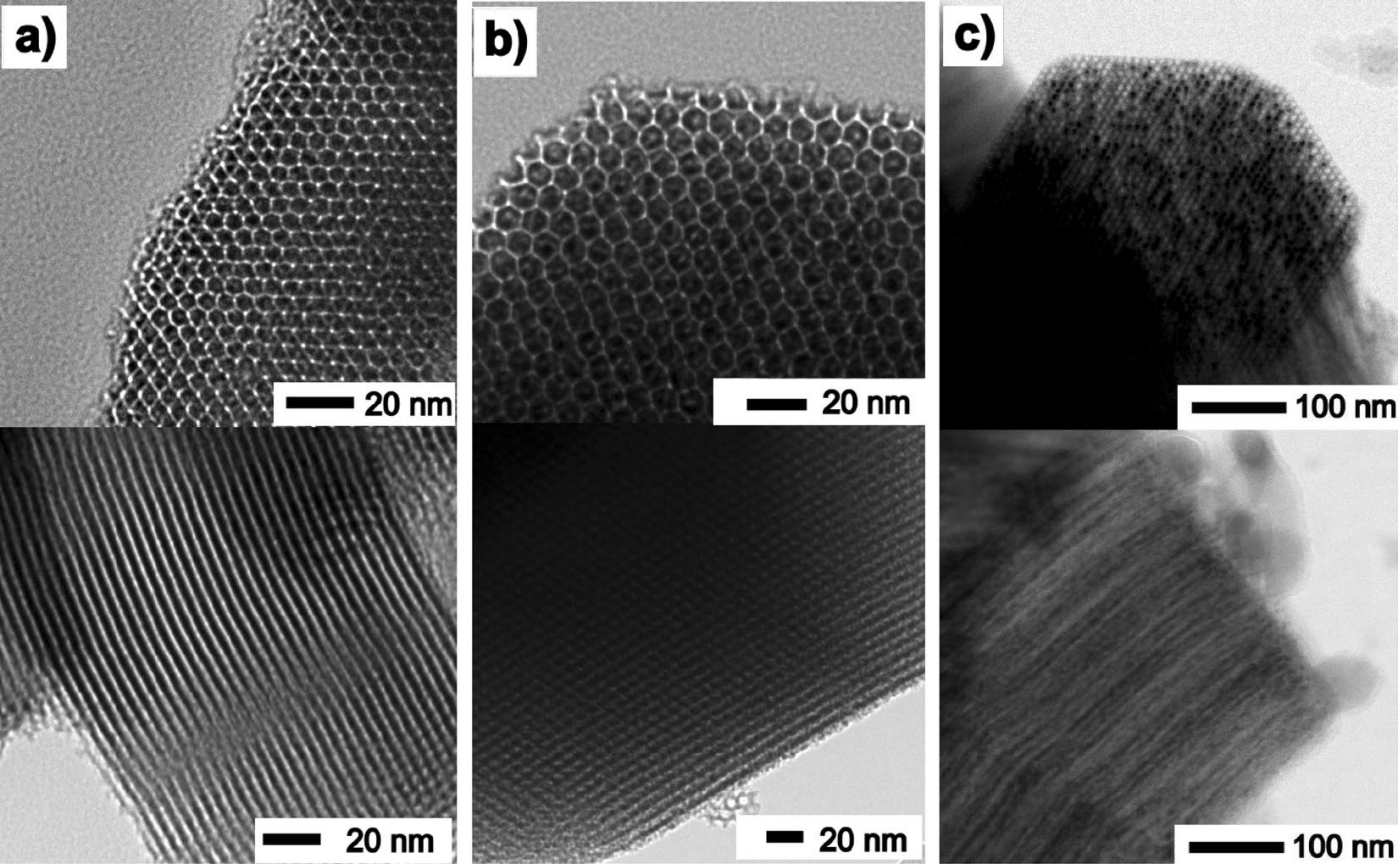
HRTEM image of Fe_2_O_3_@SBA-15 nanocomposite series. (**a**) Blank SBA-15, (**b**) Fe_2_O_3_@SBA-15 - low concentration of nanoparticles, (**c**) Fe_2_O_3_@SBA-15 - high concentration of nanoparticles. Perpendicular (upper raw) and longitudinal (bottom raw) views to hexagonal axis.

Nanocomposites examined in the study have been prepared in the manner schematically described in the Fig. 1 following the nanocasting method. Briefly, a blank SBA-15 silica matrix was synthesized by conventional procedure using triblock copolymer Pluronic P123 ((EO)20(PO)70(EO)20)) and tetraethylortosilicate (Si(OC2H5)4, TEOS) in acidic conditions (HCl). Subsequently, the matrix was used as a hard template while modified by iron oxides via wet-impregnation. Four SBA-15 systems of typical structure shown in Figs 1 and 2, with increasing concentration of hematite nanoparticles (iron precursor concentrations 0.01 M, 0.1 M, 0.5 M and 4 M) have been prepared and denoted as Fe_2_O_3_@SBA-15 (1), (2), (3) and (4), respectively. Detailed information on these systems can be found elsewhere^22^. Despite the fact that methods for the fabrication of regular hollow matrices are well established, subsequent matrix modification is a process that is very sensitive to a number of factors. Therefore, the final products have been examined experimentally. In the case of Fe_2_O_3_@SBA-15 nanocomposites scrutinized in this study, an image of inner structure comprises regular ordered matrix with hexagonal symmetry (P6mm) and polydisperse spheric NPs of *α*-iron oxide (hematite) loaded in the longitudinal pores of diameter approximately 8 nm, Fig. 1. The conception has been built assuming wealth of experimental support that has already been reported elsewhere^14,22^.

Figure 2 shows the surface structures of selected Fe_2_O_3_@SBA-15 systems, typical representants of the examined series, captured by HRTEM. One can clearly recognize NPs (dark spheres, e.g. Fig. 2c) loaded in the regular pores organized in perfect hexagonal symmetry. The NPs’ presence in all samples from the series has been corroborated by the means of energy dispersive X-ray spectroscopy (EDS)^14^. The abundance of NPs in the matrices has been found corresponding to the metallic ion precursor concentration.

**Figure 3:**
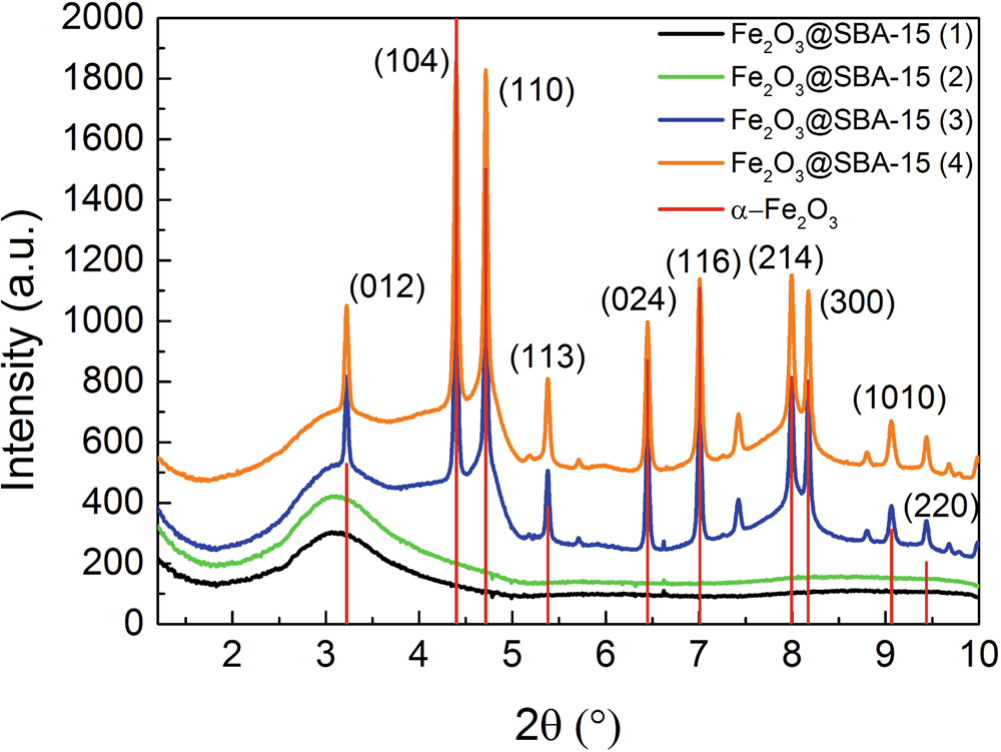
High energy XRD patterns HE-XRD of examined Fe_2_O_3_@SBA-15 nanocomposite series, reference reflection for *α*-Fe_2_O_3_ (hematite, space group R$\overline{3}c$ (No. 167), JCPDS No. 86-0550).

The XRD patterns of the sample series are presented in the Fig. 3. There, amorphous character of hosting matrix is evident by the presence of broad peak at 2*θ* = 3.09°. Crystalline *α*-Fe_2_O_3_ phase (hematite, space group R$\overline{3}c$ no. 167, JCPDS no. 86–0550) attributed to NPs has been observed only in the systems with higher precursor concentrations (samples (3) and (4)).

A putative inconsistence between EDS and XRD data has been profoundly discussed and explained elsewhere^14^. It has been concluded (in consistence with magnetic measurements) that XRD signal of NPs embedded in the pores is hidden by the scattering of amorphous SiO_2_ at the low concentrations of NPs. On the other hand, the crystalline phase is well resolved in the case of more populated iron oxide particles in the pores as well as those developed at the surface of matrix (Fe_2_O_3_@SBA-15 (3), (4)). The occupation of the matrix pores by hematite NPs has thus been confirmed unequivocally, although the information on the structural characteristics of these objects is absent. This is the point, where feasible properties of neutrons can be exploited. Contrary to X-rays, they are able to penetrate the body of the composite, and due to a significant contrast between scattering from matrix and that from NPs, they can reveal an inner structure of the system. SANS data obtained for the series of Fe_2_O_3_@SBA-15 nanocomposites examined in this study show clearly two general features that we will analyze and discuss below.

## Model Description

In order to study the internal structure of examined composite systems, we adopted a fundamental theoretical model describing SANS from hollow SBA-15 matrix as reported previously^23–25^. We extend the model by further imposing additional term responsible for scattering from individual spherical objects that represent the nanoparticles embedded, along with taking into account the size distributions of various structural features of the nanocomposite.

General formula of the model comprises four terms describing in sum the experimentally measured SANS intensity as a function of momentum transfer *q*

1 $$I(q)=KS(q){P}_{c}(q)+{I}_{diff}(q)+{I}_{i}+{K}_{g}{P}_{g}(q).$$

The first term combines the structure factor *S*(*q*) of ordered 2D hexagonal lattice with the squared form factor of cylindrical pores *P*_*c*_(*q*) that correspond to the underlying SBA-15 matrix^23,24^. It is assumed that pores are of a circular cross-section while polydisperse in size. Scaling constant *K* weighs the ordered structure contribution to the total scattering, as well as it accounts for experimental setup conditions like the incident beam flux, solid angle of the detector, and the detector efficiency. *I*_*diff*_(*q*) represents the diffuse scattering due to micropores or other inhomogeneities of the matrix, and *I*_*i*_ is the incoherent scattering which is treated as a constant in our model. The last term then characterizes the scattering from randomly distributed polydisperse spheres with constant *K*_*g*_ scaling its contribution to the overall intensity. In this study, we refer to the model described by Eq. 1 as a simple model.

Since it turned out that simple model is not sufficient for fitting experimental data from all the examined system series, we have suggested some further modifications. We append another term *K*_*g*2_*P*_*g*2_(*q*) with an identical mathematical formula to that of *K*_*g*_*P*_*g*_(*q*) in order to describe the scattering from polydisperse spheres of different average radius and standard deviation than those used in te *K*_*g*_*P*_*g*_(*q*) case. By this extension, we obtain bimodal model which can be applied to the systems with nanoparticle distributions of two different sizes.

Finally, another modification of the simple model has been done due to a possibility of creation nanorods instead of spheres inside the longitudinal matrix pores. We substitute the last term *K*_*g*_*P*_*g*_(*q*) by the expression describing scattering from polydisperse rods (cylinders) *K*_*rod*_*P*_*rod*_^*tot*^(*q*).

In all variants, the system is considered fully isotropic due to the powder form of SBA-15 matrix with a grain size of about 200–500 nm. The spherically averaged structure factor of an ideal undistorted lattice is given by^26^

2 $$S(q)\propto \frac{1}{{q}^{2}}\sum _{hk}\,{m}_{hk}{L}_{hk}(q),$$

where 1/*q*^2^ is the Lorentz factor for a powder sample, *m*_*hk*_ is the peak multiplicity for Miller indices *h*, *k* (*m*_*h*0_ = *m*_*hh*_ = 6, otherwise *m*_*hk*_ = 12), and *L*_*hk*_(*q*) is the peak shape function at a position

3 $${q}_{hk}=\frac{4\pi }{{a}_{0}\sqrt{3}}\sqrt{{h}^{2}+{k}^{2}+hk},$$

with *a*_0_ being the lattice parameter. We assume Gaussian peaks with standard deviation *σ*_*hk*_ defined by

4 $${L}_{hk}(q)=\frac{1}{\sqrt{2\pi }{\sigma }_{hk}}\exp -\frac{1}{2}{(\frac{q-{q}_{hk}}{\sigma })}^{2}.$$

The form factor for cylindrical pores of radius *R* and scattering length density *ρ*_*c*_ surrounded by homogeneous matrix of *ρ* is derived as^24^

5 $${F}_{c}(q,R)=2\pi (\rho -{\rho }_{c}){R}^{2}\frac{{J}_{1}(qR)}{qR},$$

where *J*_1_ is the Bessel function of the first order

6 $${J}_{1}(q,R)=\mathop{\sum }\limits_{m=0}^{\infty }\,\frac{{(-1)}^{m}}{m!(m+1)!}{(\frac{qR}{2})}^{2m+1}.$$

We expand this term by accommodating the polydispersity of cylinder radii assembly following Gaussian distribution with standard deviation *σ*_*c*_. Since *F*_*c*_(*q*) depends significantly on the size of the cylinder, we convolute it with the size distribution function by attributing appropriate weight coefficients *w*(*R*_*i*_). The factors *w*(*R*_*i*_) correspond to the relative population of pores with radius *R*_*i*_ via Gaussian function. Therefore, the total contribution of this term is the weighted sum of |*F*_*c*_(*q*, *R*_*i*_)|^2^ calculated by the means of Eqs 5 and 6 for each *R*_*i*_ value, that can be expressed numerically as

7 $${P}_{c}(q)=\mathop{\sum }\limits_{i=1}^{\infty }\,w({R}_{i})|{F}_{c}(q,{R}_{i}){|}^{2}.$$

Next term in the Eq. 1 corresponds to the diffuse scattering *I*_*diff*_(*q*) that originates predominantly from silica matrix. As SBA-15 consists of particles of submicrometer diameter, Porod scattering *I*_*P*_(*q*) from the outer surface of the particles will be prevalent in the *q* range well below *q*_10_, and diffuse scattering *I*_*D*_(*q*) due to micropores and other inhomogeneities of the matrix will contribute in a *q* range extending to well above *q*_10_. This contribution can then be modelled by^24^

8 $${I}_{diff}={I}_{P}(q)+{I}_{D}(q)=\frac{{A}_{P}}{{q}^{4}}+\frac{{A}_{D}}{{(1+{\gamma }^{2}{q}^{2})}^{2}},$$

where *A*_*P*_, *A*_*D*_ and *γ* are constants.

While the previous terms represent the model of hollow silica SBA-15 matrix, the last term in the Eq. 1 describes the scattering from randomly distributed polydisperse non-interacting nanoparticles. The particle factor *P*_g_^*^(*q*, *R*) of individual sphere with radius *R* and scattering length density *ρ*_*g*_ situated in medium having *ρ*_0_ is defined as^27^

9 $${P}_{g}^{\ast }(q,R)=\frac{{C}_{1}}{V}{(\frac{3V({\rho }_{g}-{\rho }_{0})(sin(qR)-qRcos(qR))}{{(qR)}^{3}})}^{2}+{C}_{2},$$

where *C*_1_, *C*_2_ are the scaling and background constants, respectively, and *V* the volume of the sphere. By analogy to the case of cylinders, we attributed Gaussian distribution characterized by *σ*_*g*_ to the relative population of the nanoparticles with radius *R*_*i*_, and we model their total contribution by the function

10 $${P}_{g}(q)=\mathop{\sum }\limits_{i=1}^{\infty }\,w({R}_{i}){P}_{g}^{\ast }(q,{R}_{i}).$$

In the alteration that supposes the nanorods instead of spheres, the scattering from nanoparticles is modeled by assuming the form factor for right circular cylinder with uniform scattering length density *ρ*_*rod*_^27^

11 $${F}_{rod}(q)=2({\rho }_{rod}-{\rho }_{0}){V}_{rod}sin(qLcos\alpha /2)/(qLcos\alpha /2)\frac{{J}_{1}(qr\,sin\alpha )}{qr\,sin\alpha },$$

where *L*, *r* and *V*_*rod*_ = *πr*^2^*L* are the length, radius and volume of the cylinder, respectively. Alpha is defined as the angle between the cylinder axis and the scattering vector *q*. The integral over *α* averages the form factor over all possible orientations of the cylinder with respect to *q*. The output of the 1D scattering intensity function for randomly oriented cylinders is then given by^27^

12 $${P}_{rod}(q)=\frac{{C}_{a}}{{V}_{rod}}{\int }_{0}^{\pi /2}\,{F}_{rod}^{2}(q,\alpha )sin\,\alpha d\alpha +{C}_{b},$$

where scaling and background constants are denoted as *C*_*a*_, *C*_*b*_, respectively. Again, Gaussian polydispersity of cylinder lengths and radii was taken into account leading to total scattering contribution from the system of rods with lengths *L*_*i*_ and radii *r*_*j*_ and their corresponding standard deviations *σ*_*L*_, *σ*_*r*_

13 $${P}_{rod}^{tot}(q)=\mathop{\sum }\limits_{i=1}^{\infty }\,\mathop{\sum }\limits_{j=1}^{\infty }\,{w}_{L}({L}_{i},{\sigma }_{L}){w}_{r}({r}_{j},{\sigma }_{r}){P}_{rod}(q,{L}_{i},{r}_{j}).$$

The key feature of the proposed model is the ability to treat separately the nanocomposite as a system of hollow matrix and individual non-interacting nanostructures. Optimizing the scaling constants *K*, *K*_*g*_, *K*_*g*2_ and *K*_*rod*_ via fitting the appropriate model to experimental data, one can asses the contribution of particular subsystem, and infer the concentration of nanoparticles embedded in the matrix. Adjusting the fitting parameters, it is also possible to establish crucial structural characteristics of all subsystems, including the shape and size distributions of these structures and/or the lattice parameter of ordered pores.

## Results and Discussion

### Model application

Seminal structural examination^14,22,28^ of all the nanocomposite systems of interest (Fe_2_O_3_@SBA-15 (1–4)) revealed previously fairly good ordered pores of silica matrix of p6mm symmetry with average pore diameter *R*_*c*_ ~ 7 nm and mutual center-to-center distance *a*_0_ ~ 10 nm. The presence of Fe_2_O_3_ phase has also been unequivocally confirmed in all the systems, though its characterization was not possible in the case of small concentrations of NPs. We therefore extend these studies by the means of SANS measurements and their evaluation employing the above proposed model.

**Figure 4:**
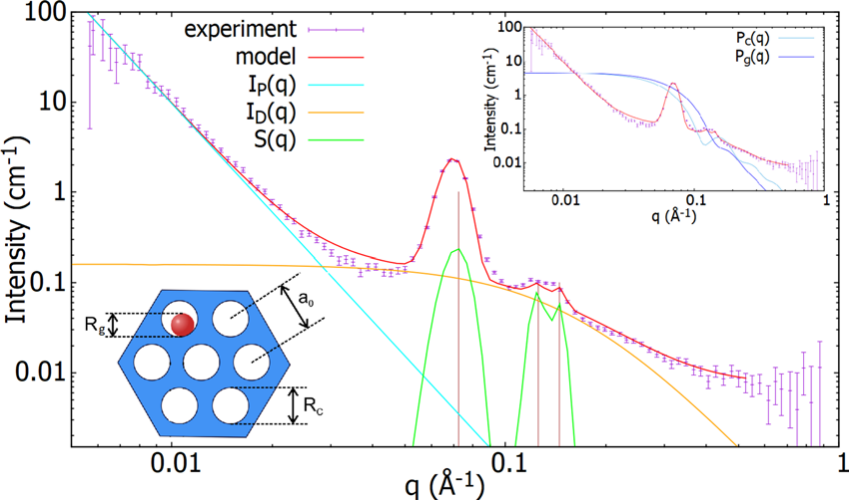
Fit of the model (Eq. 1) to the experimental SANS data corresponding to nanocomposite Fe_2_O_3_@SBA-15 (1) with very sparse occupation of the pores by nanoparticles. (inset at the top right) Dark and light blue lines represent rescaled (facilitating the comparison) contributions of the polydisperse spheres (nanoparticles) and cylinders (ordered pores), respectively, to the total SANS intensity. (inset at the bottom left) The schematics of nanocomposite with structural parameters allowed to adjust during the model fitting to the data.

The first system we scrutinize is the nanocomposite with the lowest nanoparticle concentration, Fe_2_O_3_@SBA-15 (1). Due to a sparse occupation of pores by NPs, the system can be considered as hollow SBA-15 matrix with only a minor contribution of signal originating from the spheres to the total SANS intensity. Adjusting the model to fit the experimental data, we establish at first the parameters characteristic to the matrix. Figure 4 shows the best fit (red curve) of Eq. 1 to the Fe_2_O_3_@SBA-15 (1) data along with the contributions to the total intensity originating from particular features of the matrix. Parameters of the fit are listed in Table 1.

**Table 1:** List of parameters corresponding to the best fits of appropriate model to examined nanocomposites’ SANS data.

System	Model	*R*_*g*_ (Å)	*σ*_*g*_ (Å)	*R*_*g*2_ (Å)	*σ*_*g*2_ (Å)	*A*_*p*_ (Å^−4^)	*A*_*D*_	*γ*_*i*_ (Å)	*I*_*i*_ (*cm*^−1^)	*K*	*K*_*g*_	*K*_*g*2_
Fe_2_O_3_@SBA-15 (1)	simple model	30	5	—	—	0.96	0.16	6.14	7.49 × 10^−3^	3	2.55	—
Fe_2_O_3_@SBA-15 (2)	simple model	32	5	—	—	1.38	0.18	6.01	6.72 × 10^−3^	3.34	6.32 × 10^4^	—
Fe_2_O_3_@SBA-15 (3)	bimodal model	35	7	100	38	4.30	1.99	14.18	1.16 × 10^−2^	1.12	8.27 × 10^5^	2.35 × 10^6^
Fe_2_O_3_@SBA-15 (4)	bimodal model	35	7	100	38	6.57	0.65	8.82	1.01 × 10^−2^	0.48	2.06 × 10^6^	1.01 × 10^7^

Employing the model, average pore center-to-center distance has been established to *a*_0_ = 10 nm (*σ*_*a*0_ = 5.8 × 10^−4^ nm) and the radii of pore cylinders to *R*_*c*_ = 4 nm (*σ*_*c*_ = 0.5 nm). These values are in excellent accordance with the results of experimental studies of the composites mentioned in the *Experimental support* section. Low standard deviations of structural features point to the fairly good ordering and regular shape of cylindrical pores of prepared SBA-15 matrix. We therefore adopt these values to the rest of systems with higher particle’s concentrations by maintaining them fixed while fitting the data.

Further, we would like to point out the scaling constants *K* and *K*_*g*_, that already in the case of the system (1) - i.e. the lowest particle concentration - are of the same order of magnitude. As it was mentioned in the *Model description* section, the ratio between the two constants refers to the weight of the contributions of particular feature to the total SANS intensity. Hence, one can infer the degree of particle concentration in particular nanocomposite by comparison of *K* and *K*_*g*_ and obtain the structural characteristics of NPs. The best results for this system is obtained while modeling the NPs with spheres of radii *R*_*g*_ = 3 nm (*σ*_*g*_ = 0.5 nm).

**Figure 5:**
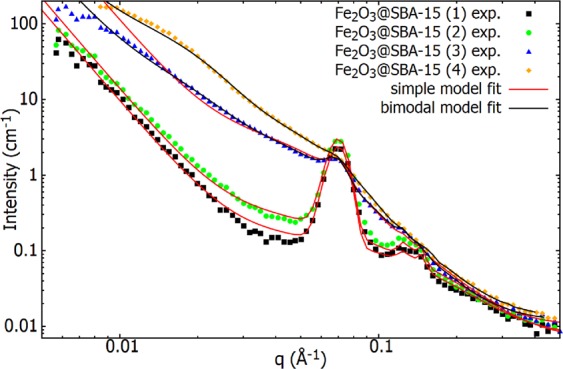
The best fits of the model to SANS experimental data corresponding to series of Fe_2_O_3_@SBA-15 nanocomposites of different nanoparticle concentrations. Red lines correspond to simple model fits (Eq. 1), while black lines represent bimodal model fits. Parameters employed in the fits are listed in the Table 1.

Figure 5 summarizes the fits of Eq. 1 (red lines) to the experimental SANS data of all the systems studied. Gradual enhancement of the SANS intensity with particle concentration increase is apparent at *q* < 0.1 Å^−1^. According to to the model, this phenomenon can be ascribed to a progressive occupation of pores by NPs. Looking closely at the shape of the curve representing the scattering from polydisperse spheres (Eqs 9, 10, and Fig. 4 (inset)) one can notice its noncommittal variance for *q* < 0.05 Å^−1^ followed by a sudden sharp descent. Indeed, a growth and shape of the shoulder in the range 0.01 < *q* < 0.1 Å^−1^ can be appropriately modeled by tuning the contribution of the last term in Eq. 1 to the total SANS intensity by the means of scaling the constant *K*_*g*_. When comparing system (1) fit to the fits corresponding to systems (2) and (3), where significant amounts of NPs occupy the pores, only the negligible changes can be seen in the parameters determining matrix structural characteristics (*A*_*p*_, *A*_*D*_, *γ*_*i*_ in Table 1). On the contrary, abrupt increase of *K*_*g*_ (up to five orders of magnitude) related to the concentration of NPs can be regarded as the evidence for the elevated amount of polydisperse spheres distributed in the body of the matrix.

Despite of rather good accordance between the model (i.e. simple model) and experimental data for the systems (1) and (2), significant discrepancies become apparent in the cases of nanocomposite with high abundance of NPs, Fe_2_O_3_@SBA-15 (3) and (4). There, as a consequence of different scenario during the matrix modification, the presence of significant fraction of Fe_2_O_3_ structures larger than pore diameter has been documented previously^14^. Large NPs of different shapes have been observed directly on the surface of the nanocomposite (4), while the development of nanorods instead of globular NPs within the longitudinal pores can not be excluded apriori either. Hence, at first we attempted to substitute the last term in the Eq. 1 for the term representing scattering from individual polydisperse Fe_2_O_3_ nanorods of various lengths. The series of modified model fits to experimental data of systems (3) and (4) however did not provide better results when compared to the fits assuming polydisperse spheres (not shown). The best fits were obtained for the rods with the sizes similar in both dimensions (i.e. *L* = 2*R*_*c*_). Thus, we concluded that the objects embedded in the matrix pores are rather spherical than tubular.

Further, we have tried to tune the model by taking into account additional term in original Eq. 1, describing scattering from system of randomly distributed polydisperse spheres of radii significantly larger than the radius of pore (i.e. bimodal particle size distribution). This improved significantly a model correlation with experimental data as shown in Fig. 5 (black lines) particularly in the case of system (4). The result is in a good agreement with the observation of larger nanoparticles on surface of the composite (4)^14^. Although the fraction of large particles in the composite is minor, according to Eq. 9 they scatter proportionally to their size. Thus, their contribution to the total SANS signal is not negligible. Based on the comparisons between simple and bimodal model fits to the data (3) and (4) (Fig. 5), we conclude the presence of a portion of larger NPs on the surface of these nanocomposite systems.

In order to challenge the conclusions on nanoparticle size distribution, we have compared the results to the data derived from magnetization measurements. Since our composites with higher NP concentrations clearly display the hallmarks of superparamagnetism^14,22^, one can employ modified Langevin law^29^ to estimate the average magnetic moment and correspondinge volume of the particle. The best fit (not shown) to the magnetization experimental data collected at *T* = 300 K as a function of applied magnetic field (up to 5 T) was obtained for composite (3) assuming log-normal NP distribution. Median nanoparticle diameter 7.9 nm (*σ*_*mag*_ = 1.32 nm) extracted from the analysis was found in a good agreement with the one determined by SANS modeling, though slightly higher. We would like to note that the method applied is indirect and due to magnetic peculiarities inherent to fine nanoparticle systems in general, one has to be very careful with the interpretation of the results. As it has been demonstrated by Brice-Profeta *et al*.^30^ in the case of *γ*-Fe_2_O_3_ nanoparticle system, magnetic volume of NP is smaller than the one observed by HRTEM. This well known phenomenon is enhanced in the case of fine particles and stems mainly from lattice irregularities at the surface of NP, leading then to spin canting. This would appear in contradiction with our observations when comparing the diameter of NP ~ 7 nm obtained from SANS. However, the study by Li *et al*.^31^ performed on series of Fe_3_O_4_ nanoparticle systems reveals the relationship between average particle size and crystallite size. It has been shown that NPs have a single crystalline structure (indicating single domain magnetic structure) up to a certain diameter. Furthermore, sphere-like NPs show crystalline size much smaller than the particle diameter, and also much smaller than those of the cube-like NPs. They concluded that sphere-like NPs commonly consist of agglomerates of variously sized cubic NPs. In the context of these findings we discuss our results as follows. In our composite (3), average *α*-Fe_2_O_3_ crystalline size 6.5 nm was determined from XRD data by means of Scherrer formula^22^. This suggests a scenario, in which nanoparticles in the pores are of single domain structure, while those at the surface of the nanocomposite, although having significantly larger sizes (HRTEM, SANS), consist of smaller variously sized NPs. At the temperature 300 K, where the NP subsystem of investigated nanocomposite is unambiguously in superparamagnetic state, the volume of single domain crystalline accounts for magnetic volume of the particle. This would explain the fact that log-normal instead of Gaussian bimodal particle size distribution (SANS) fits better the magnetization data. A more detailed analysis of the SANS results in terms of magnetic properties of the composite series will be reported elsewhere^22^.

In summary, the data analysis employing our model is in line with the results obtained for examined system series by means of different experimental methods. In addition it provides further support for the hypothesis^14^ on the existence of critical ion concentration when modifying the matrix utilizing wet impregnation. Exceeding that limit, process of nanoparticle formation takes a different path leading to expelling the particles out of pores onto the matrix surface where their further growth is not confined. More importantly, the application of our models and mutual comparison revealed the actual shape of the structures embedded in the body of the SBA-15 matrix. Speculations on nanowires or nanorods formation instead of spherical objects were ruled out. As it has been demonstrated, the conception of proposed model is based on superposition principle and it allows for addition or substitution of systems’ fundamental characteristics, being a versatile tool for scrutinizing nanocomposites with SBA-15 matrix by means of SANS.

## Conclusions

Investigations of Fe_2_O_3_@SBA-15 nanocomposite series utilizing SANS provided a wealth of results. Firstly, the presence of iron oxide NPs buried in the body of silica matrix was confirmed. Secondly, versatile model of SANS intensity dependence revealed particular features corresponding to hollow matrix and system of polydisperse spheres. By adjusting the structure parameters, information on nanocomposite structural properties was derived. The values of crucial characteristics (pore and nanoparticle size distribution, nanoparticle shape and abundance) were found in good accordance with supporting experimental data. Finaly, indirect evidence in favor of the hypothesis on different scenario of matrix modification process by means of precursor with higher metallic ion concentrations was gathered. The superposition principle employed in the model conception allows for its extension to variety of further systems.
